# Hematotoxicity and Nephrotoxicity in Prostate Cancer Patients Undergoing Radioligand Therapy with [^177^Lu]Lu-PSMA I&T

**DOI:** 10.3390/cancers14030647

**Published:** 2022-01-27

**Authors:** Philipp E. Hartrampf, Franz-Xaver Weinzierl, Sebastian E. Serfling, Martin G. Pomper, Steven P. Rowe, Takahiro Higuchi, Anna Katharina Seitz, Hubert Kübler, Andreas K. Buck, Rudolf A. Werner

**Affiliations:** 1 Department of Nuclear Medicine, University Hospital Wuerzburg, Oberdürrbacherstraße 6, 97080 Wurzburg, Germany; franz-xaver.weinzierl@stud-mail.uni-wuerzburg.de (F.-X.W.); serfling_s1@ukw.de (S.E.S.); thiguchi@me.com (T.H.); buck_a@ukw.de (A.K.B.); werner_r1@ukw.de (R.A.W.); 2 The Russell H Morgan Department of Radiology and Radiological Science, Johns Hopkins University School of Medicine, 601 N Caroline Str., Baltimore, MD 21205, USA; mpomper@jhmi.edu (M.G.P.); srowe8@jhmi.edu (S.P.R.); 3 Graduate School of Medicine, Dentistry and Pharmaceutical Sciences, Okayama University, Okayama 700-8530, Japan; 4 Department of Urology and Paediatric Urology, University Hospital Wuerzburg, Oberdürrbacherstraße 6, 97080 Wurzburg, Germany; seitz_a3@ukw.de (A.K.S.); kuebler_h@ukw.de (H.K.)

**Keywords:** PSMA, radioligand therapy, RLT, ^177^Lu, nephrotoxicity, hematotoxicity, CTCAE

## Abstract

**Simple Summary:**

Radioligand therapy (RLT) with prostate-specific membrane antigen (PSMA)-directed agents has shown remarkable results in patients with advanced prostate cancer. Our objective was to provide data on the side effect profile of PSMA-directed RLT using the therapeutic radiotracer [^177^Lu]Lu-PSMA I&T. We evaluated patients with castration-resistant metastatic prostate cancer treated with at least three cycles of [^177^Lu]Lu-PSMA I&T. A substantial fraction of the patients already had impaired renal function and/or reduced white blood cell counts at baseline, but the degree of nephrotoxicity or hematotoxicity under RLT was low. No severe toxicities occurred under RLT.

**Abstract:**

(1) Background: Prostate-specific membrane antigen (PSMA)-directed radioligand therapy (RLT) has shown remarkable results in patients with advanced prostate cancer. We aimed to evaluate the toxicity profile of the PSMA ligand [^177^Lu]Lu-PSMA I&T. (2) Methods: 49 patients with metastatic, castration-resistant prostate cancer treated with at least three cycles of [^177^Lu]Lu-PSMA I&T were evaluated. Prior to and after RLT, we compared leukocytes, hemoglobin, platelet counts, and renal functional parameters (creatinine, eGFR, *n* = 49; [^99m^Tc]-MAG3-derived tubular extraction rate (TER), *n* = 42). Adverse events were classified according to the Common Terminology Criteria for Adverse Events (CTCAE) v5.0 and KDIGO Society. To identify predictive factors, we used Spearman’s rank correlation coefficient. (3) Results: A substantial fraction of the patients already showed impaired renal function and reduced leukocyte counts at baseline. Under RLT, 11/49 (22%) patients presented with nephrotoxicity CTCAE I or II according to creatinine, but 33/49 (67%) according to eGFR. Only 5/42 (13%) showed reduced TER, defined as <70% of the age-adjusted mean normal values. Of all renal functional parameters, absolute changes of only 2% were recorded. CTCAE-based re-categorization was infrequent, with creatinine worsening from I to II in 2/49 (4.1%; GFR, 1/49 (2%)). Similar results were recorded for KDIGO (G2 to G3a, 1/49 (2%); G3a to G3b, 2/49 (4.1%)). After three cycles, follow-up eGFR correlated negatively with age (r = −0.40, *p* = 0.005) and the eGFR change with Gleason score (r = −0.35, *p* < 0.05) at baseline. Leukocytopenia CTCAE II occurred only in 1/49 (2%) (CTCAE I, 20/49 (41%)) and CTCAE I thrombocytopenia in 7/49 (14%), with an absolute decrease of 15.2% and 16.6% for leukocyte and platelet counts. Anemia CTCAE II occurred in 10/49 (20%) (CTCAE I, 36/49 (73%)) with a decrease in hemoglobin of 4.7%. (4) Conclusions: After PSMA-targeted therapy using [^177^Lu]Lu-PSMA I&T, no severe (CTCAE III/IV) toxicities occurred, thereby demonstrating that serious adverse renal or hematological events are unlikely to be a frequent phenomenon with this agent.

## 1. Introduction

In recent years, therapeutic regimens for patients with metastatic castration-resistant prostate cancer (mCRPC) have been expanded by novel drug developments, e.g., the next-generation androgen receptor signaling inhibitors abiraterone and enzalutamide [1,2], or chemotherapy with docetaxel or cabazitaxel [3,4,5]. Further advances include the introduction of prostate-specific membrane antigen (PSMA)-directed diagnostic agents and their therapeutic equivalents, which have been evaluated in prospective trials with remarkable outcomes in advanced disease [6,7,8,9,10]. Of note, mCRPC patients treated with radioligand therapy (RLT) using [^177^Lu]-PSMA only experienced mild side effects, with kidneys and bone marrow as organs at risk. For instance, a recent meta-analysis, including predominantly retrospective data of 24 studies (20 conducted with [^177^Lu]Lu-PSMA-617 and only three with [^177^Lu]Lu-PSMA I&T), showed that hematotoxicity is not a frequent phenomenon. Nephropathy was also reported in only 13% of the patients with very few grade III/IV toxicities [11].

Those retrospective reports have been further corroborated in a prospective setting, in particular by using [^177^Lu]Lu-PSMA-617. For instance, the recently published Lu-PSMA trial reported on grade III/IV lymphopenia, thrombopenia, anemia, and neutropenia in up to 32% of the cases, whereas for the kidneys, only grade I/II events were reported in 10% of the treated patients [8]. In the TheraP-trial investigating [^177^Lu]Lu-PSMA-617 vs. cabazitaxel, grade III/IV adverse events occurred in 33% of 98 patients in the RLT arm. Of note, grade III/IV thrombocytopenia, but not neutropenia, was more common with [^177^Lu]Lu-PSMA-617 when compared to cabazitaxel [9]. In the VISION trial, the largest prospective cohort studied to date, 52.7% of the patients showed events of ≥grade III under RLT. Again, the toxicity profile was predominantly characterized by anemia, thrombocytopenia, and lymphopenia, whereas for the kidneys, no new or unexpected safety concerns were noted [10,12].

All of those studies used [^177^Lu]Lu-PSMA-617, while data on the toxicity profile of [^177^Lu]Lu-PSMA I&T are scarce. Heck et al. presented the largest cohort of patients (*n* = 100) treated with [^177^Lu]Lu-PSMA I&T and showed that treatment-related toxicities were mainly hematologic, including grade III/IV toxicities, such as anemia (9%), thrombocytopenia (4%), and neutropenia (6%). Serious (≥grade III) non-hematologic toxicities, in particular for the kidneys, were not observed [13].

To date, it has generally been assumed that [^177^Lu]Lu-PSMA I&T and [^177^Lu]Lu-PSMA-617 are comparable in their efficacy and safety [11,14]. Given the imbalance of reliable data on [^177^Lu]Lu-PSMA I&T relative to -617, we aimed to corroborate the favorable safety profile of [^177^Lu]Lu-PSMA I&T in the present mono-centric study by evaluating 118 patients treated with RLT with a focus on nephrotoxicity and hematotoxicity.

## 2. Materials and Methods

### 2.1. Patient Cohort

Between June 2015 and May 2021, 118 consecutive patients who started their treatment with PSMA-directed RLT for mCRPC were screened for eligibility. Inclusion criteria for toxicity assessment were patients undergoing RLT for at least three cycles, with available follow-up 6–8 weeks after the third cycle or re-admission for the fourth cycle (with laboratory assessment at day 1 prior to administration of [^177^Lu]Lu-PSMA I&T). An extended follow-up cohort included patients with at least 5 cycles of RLT. Ethical review and approval were waived for this study by the local Ethics Committee due to the retrospective character of the study (waiver no. 20210422 04).

### 2.2. Pre-Therapy Work-Up

During pre-therapeutic work-up, patients received [^99m^Tc]Tc-mercaptoacetyltriglycine (MAG3) scintigraphy for the evaluation of renal function. At baseline and at every re-admission for the next cycle, blood samples were collected prior to administration of the therapeutic compound at the day of hospital admission, including creatinine level (mg/dL; norm: 0–1.17 mg/dL), estimated glomerular filtration rate (eGFR; CKD-EPI equation; lower normal limit: 90 mL/min/1.73 m^2^) and hematological parameters (platelets, hemoglobin and leukocyte counts). Blood collection for blood cell count was performed using di-potassium-ethylenediaminetetraacetic acid (EDTA) tubes (Sarstedt, Nuembrecht, Germany) and analyzed using a fully automated modular analyzer (Sysmex XN-9000, Kobe, Japan). Blood collection for creatinine and eGFR was performed using serum-gel tubes (Sarstedt, Nuembrecht, Germany) and analyzed using a fully automated modular analyzer (Roche Cobas, Basel, Switzerland).

### 2.3. Treatment Protocol

Radiosynthesis of [^177^Lu]Lu-PSMA I&T was performed by adding a solution of 150 µg PSMA I&T (Scintomics Molecular, Applied Theranostics Technologies GmbH, Fuerstenfeldbruck, Germany) and 7 µg gentisic acid in 525 µL of a 0.4 M sodium acetate buffer solution (pH 5.2) to a vial containing 6–7 GBq [^177^Lu]LuCl_3_ in 200 µL 0.04 M HCl (EndolucinBeta^®^, ITM Medical Isotopes GmbH, Garching, Germany) and heating the vial in a heating block for 35 min at 100 °C. After cooling, the solution was diluted with saline and passed through a sterile filter (0.22 mm). Every single batch of [^177^Lu]Lu-PSMA I&T was tested for radiochemical purity by gradient high-performance liquid chromatography and thin-layer chromatography. Furthermore, pH-value and bubble point were determined before releasing the product.

The standard PSMA RLT protocol consisted of infusion of 6.0 GBq of the radioligand every 8 weeks with up to a maximum of 8 cycles depending on response to treatment. In patients with significantly impaired renal function, the activity administered was decreased by approximately 20%. A total of 30 min before the administration of the RLT, an infusion of 1000–2000 mL of 0.9% sodium chloride (NaCl) solution was started and maintained until 4 h after the injection. No furosemide was administered.

### 2.4. Toxicity Assessment

Medical records were used for assessment of potential risk factors for renal disease (e.g., age, hypertension, diabetes mellitus, and pre-existing renal disease) and potential nephrotoxic medication (analgesics, antihypertensive medication, lipid-lowering medication, and diuretics). Following discharge after each therapy cycle, the laboratory tests were continued at 2-week intervals in an outpatient clinic. Results were sent to our department for a period of at least 8–12 weeks in accordance with European Association of Nuclear Medicine procedure guidelines [14]. To standardize the time points of toxicity assessment, we analyzed renal function and blood parameters on the day of admission for the next cycle of therapy. In case of termination after three cycles, parameters 8 (±2) weeks after the last cycle were analyzed. Renal scintigraphy was repeated after every two cycles of RLT.

Renal function regarding creatinine levels and eGFR was graded according to the Common Terminology Criteria for Adverse Events (CTCAE v5.0) [15], and we also classified the renal function according to Kidney Disease: Improving Global Outcomes (KDIGO) society [16]. Definition of a nephrotoxic adverse event also followed the KDIGO society:

1. “Decline in GFR category (≥90 (G1), 60–89 (G2), 45–59 (G3a), 30–44 (G3b), 15–29 (G4), <15 (G5) mL/min/1.73 m^2^). A certain drop in eGFR is defined as a drop in GFR category accompanied by a 25% or greater drop in eGFR from baseline.
2. Rapid progression is defined as a sustained decline in eGFR of more than 5 mL/min/1.73 m^2^/year” [17].

For the interpretation of the tubular extraction rate (TER) from renal scintigraphy, we used a threshold of 70% of the age-dependent normal values to classify an event.

For the grading of adverse events regarding leukocytes and platelets, we again used CTCAE v5.0.

### 2.5. Statistical Analysis

Statistical analyses were performed using GraphPad Prism version 9.0.2 for Windows (GraphPad Software, San Diego, CA USA). Descriptive data are presented as mean  ±  standard deviation. Since not all parameters were normally distributed, comparisons between baseline and follow-up values were performed using Wilcoxon signed-rank test. For correlation analysis, a Spearman’s rank correlation coefficient was calculated. A *p*-value less than 0.05 was considered statistically significant.

## 3. Results

### 3.1. Patients

Of 118 screened patients, 49 (41.5%) patients received at least 3 cycles and were included for further analysis. A total of 17 patients received additional cycles with a median of 7 cycles (range: 6–9). Starting at cycle 1, day 1, the mean timespan until cycle 2, day 1 was 8.8 ± 1.1 weeks, until cycle 3, day 1 18.0 ± 1.8 weeks, and until cycle 4, day 1 26.8 ± 2.2 weeks. The patient cohort consisted of mainly elderly men with a mean age of 71.2 ± 8.4 years. A total of 12/49 (24.5%) patients had pre-existing renal disease, 32/49 (65.3%), had arterial hypertension, 8/49 (16.3%) suffered from diabetes mellitus, and 34/49 (69.4%) were taking potential nephrotoxic medication. Previous systemic therapies for mCRPC included enzalutamide (77.6%) and abiraterone acetate (71%). Prior chemotherapy was conducted in 57.1% of the patients, and all patients had received antihormonal treatment. Initial tumor burden was mainly >10 lesions (85.7% of the patients), of which 12 patients showed a diffuse bone involvement (24.5%). The following organs systems were mainly affected: bones (93.9%), lymph nodes (71.4%), and local tumor (34.7%). Detailed characteristics can be found in Table 1.

### 3.2. Baseline Categorization of Renal Toxicity

**Figure 1:**
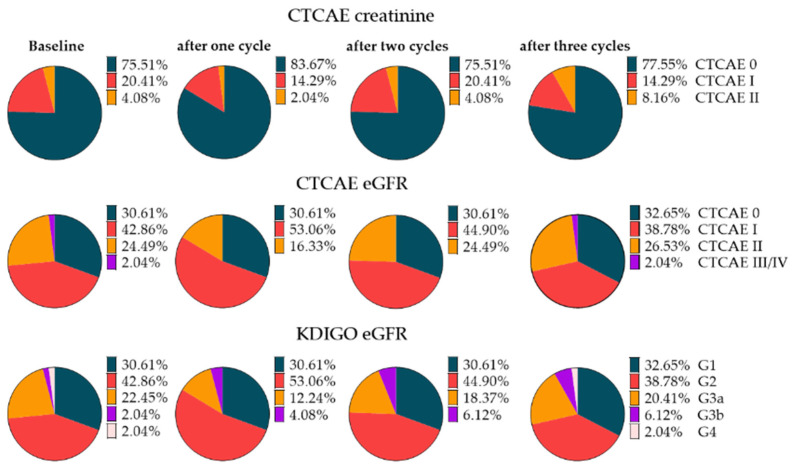
Pie charts for different classification systems for nephrotoxicity under radioligand therapy (RLT) in patients with three cycles. First two rows: Classifications according to CTCAE for creatinine and eGFR for 49 patients under RLT before and at each cycle of therapy. Last row: Classification according to KDIGO in 49 patients under RLT before and at each cycle of therapy. Regardless of which classification was used, the slices remained almost identical between subsequent cycles. eGFR = estimated glomerular filtration rate.

Before therapy initiation, 37/49 (76%) patients did not show any CTC adverse event according to creatinine serum levels, while 12/49 (24%) already showed an impaired renal function (CTCAE I and II). No grade III/IV events were recorded (Figure 1). The mean baseline creatinine level was 1.02 ± 0.33 mg/dL. According to the eGFR, 15/49 patients (31%) did not show any event, 21/49 (43%) showed CTCAE I, 12/49 (24%) showed CTCAE II, and only one patient showed CTCAE III (2%) (Figure 1). Mean baseline eGFR was 76.5 ± 20.1 mL/min/1.73 m^2^.

**Figure 2:**
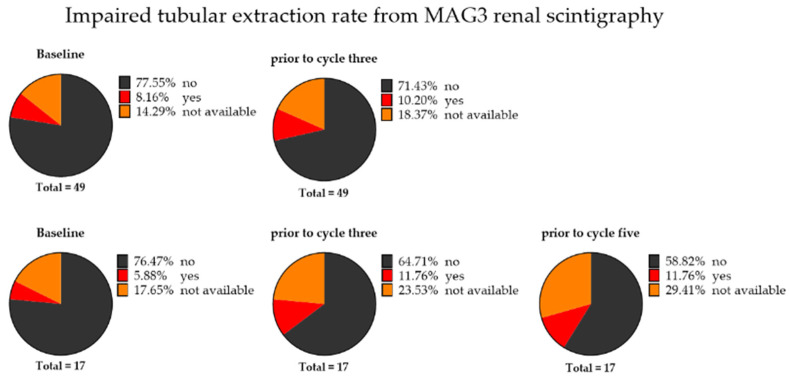
Pie charts for tubular extraction rate (TER), with patients having either impaired (yes) or no impaired kidney function (no), defined as <70% of the age-adjusted mean normal values. First row: Classifications according to TER from renal scintigraphy for 49 patients under radioligand therapy before and after two cycles of therapy. Last row: Classification according to TER from MAG3 scintigraphy for 17 patients under radioligand therapy before, after two cycles, and after four cycles of therapy. After four therapy cycles, there was an increase in patients with impaired kidney function relative to baseline.

MAG3 renal scintigraphy was available in 42 of 49 patients. Renal tubular extraction rate (TER) was normal in 38/42 patients (90%), while four patients (10%) showed reduced TER at baseline (Figure 2). The mean baseline TER was 213.3 ± 61.5 mL/min/1.73 m^2^.

Categorization according to KDIGO was as follows: G1, 15/49 (31%); G2, 21/49 (43%); G3a, 11/49 (22%); G3b, 1/49 (2%); and G4, 1/49 (2%) (Figure 1).

### 3.3. Renal Toxicity with RLT

After three cycles of RLT, 11/49 (22%) patients showed any CTCAE toxicity for creatinine and 33/49 (67%) for eGFR, respectively, but the distribution of the CTCAEs remained almost unchanged relative to baseline. According to creatinine, only two patients worsened from grade I to grade II, and according to eGFR, only one patient worsened from grade I to grade II. No new CTCAEs III or IV were recorded under ongoing RLT (Figure 1). Mean creatinine level after three cycles slightly increased from 1.02 ± 0.33 to 1.04 ± 0.37 mg/dl (+2.0%, *p* = 1.0), while eGFR slightly dropped from 76.5 ± 20.1 to 75.0 ± 21.8 mL/min/1.73 m^2^ (−2.0%, *p* = 0.72). After three cycles of RLT, the distribution of categorization according to KDIGO showed only minor changes. One patient worsened from G2 to G3a, and two patients worsened from G3a to G3b after three cycles (Figure 1).

Renal scintigraphy after the second treatment with RLT was available in 40 patients, and only five patients (13%) showed reduced TER (Figure 2). TER after three cycles slightly dropped from 213.3 ± 61.5 to 209.1 ± 56.8 mL/min/1.73 m^2^ (−2.0%, *p* = 0.83).

### 3.4. Extended Follow-Up Cohort

**Figure 3:**
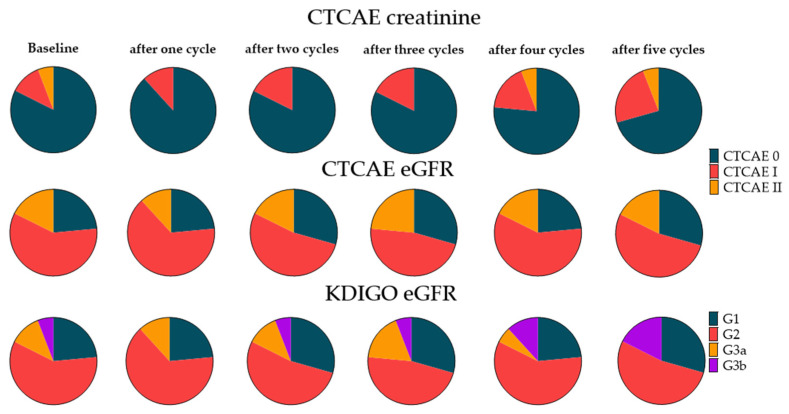
Pie charts for different classification systems for nephrotoxicity under radioligand therapy (RLT) in patients with extended follow-up. First two rows: Classifications according to CTCAE for creatinine and eGFR for 17 patients under radioligand therapy before and at each cycle of therapy. Last row: Classification according to KDIGO in 17 patients under radioligand therapy before and at each cycle of therapy. Again, regardless of which classification was used, the slices remained almost identical, even after five cycles. eGFR = estimated glomerular filtration rate. Detailed percentages can be found in the Table S1.

Extended follow-up after the 5th cycle was available for 17 patients. For creatinine, 14/17 patients (82%) did not show any CTCAE grade toxicity, 2/17 (12%) showed grade I, and 1/17 (6%) showed grade II toxicity at baseline. For eGFR at baseline, 4/17 (24%) did not show any CTCAE, 10/17 (59%) showed grade I, and 3/17 (18%) showed grade II toxicity (Figure 3). Serum creatinine levels slightly increased from 0.98 ± 0.28 to 1.01 ± 0.27 mg/dL (+3.1%, *p* = 0.33) after three cycles and up to 1.06 ± 0.34 mg/dL (+8.2%, *p* = 0.005) after five cycles.

After five cycles, two patients worsened from grade 0 to grade I toxicity according to creatinine levels. Kidney function measured with eGFR improved in one patient, with re-categorization from CTCAE I to CTCAE 0 (Figure 3). eGFR slightly dropped from 78.9 ± 18.4 to 75.0 ± 17.6 mL/min/1.73 m^2^ (−4.9%, *p* = 0.15) after three cycles, with a decrease to 72.3 ± 20.4 mL/min/1.73 m^2^ (−8.4%, *p* = 0.002) after five cycles.

Baseline categorization according to KDIGO was as follows: G1, 4/17 (24%); G2, 10/17 (59%); G3a 2/17 (12%) and G3b, 1/17 (6%). After five cycles of RLT, the KDIGO-based distribution showed only minor changes. Two patients worsened from G3a to G3b, whereas one patient moved up from G2 to G1 (Figure 3).

Renal scintigraphy was available in 14 of 17 patients and showed reduced TER at baseline in only one patient (7%). After the second cycle, two patients (of 13 available, 12%) showed impaired TER, and after the fourth cycle, another two patients (of 12 available, 12%) showed reduced TER (Figure 2). TER slightly dropped from 215.6 ± 48.1 to 212.1 ± 57.2 mL/min/1.73 m^2^ (−1.6%, *p* = 0.88) after two cycles and decreased to 179.8 ± 43.6 mL/min/1.73 m^2^ (−16.6%, *p* = 0.005) after four cycles.

### 3.5. Risk Factor Assessment for Renal Toxicity

Significant correlations were found for eGFR at baseline with follow-up eGFR after three cycles (r = 0.83, *p* < 0.001). In addition, pre-therapeutic TER correlated with pre-therapeutic (r = 0.76, *p* < 0.001) and follow-up eGFR after three cycles (r = 0.61, *p* < 0.001).

Significant inverse correlation was found for age at first cycle with eGFR at the first cycle (r = −0.44, *p* = 0.002) and with the follow-up eGFR after three cycles (r = −0.40, *p* = 0.005) as well as for Gleason score at initial diagnosis with the relative change of the eGFR after three cycles of therapy (r = −0.35, *p* < 0.05). There were no significant correlations with change in eGFR (r = −0.02; *p* = 0.91) or administered cumulative activity after three cycles (r = 0.2; *p* = 0.17).

### 3.6. Hematotoxicity with RLT

Before therapy initiation, 39/49 patients (80%) did not show any CTCAE grade leukocytopenia, while 10/49 (20%) already showed a reduced number of leukocytes (only CTCAE I). Only one subject showed reduced platelet counts (CTCAE I). For hemoglobin, 34/49 (69%) already showed grade I toxicity, 10/49 (20%) showed grade II toxicity, and one patient showed grade III toxicity. No CTCAEs III/IV were recorded. Mean leukocyte and platelet counts at baseline were 6.6 ± 2.5 * 1000/µL and 259.3 ± 79.5 * 1000/µL, respectively. Hemoglobin (Hb) was 11.5 ± 1.7 g/dL.

**Figure 4:**
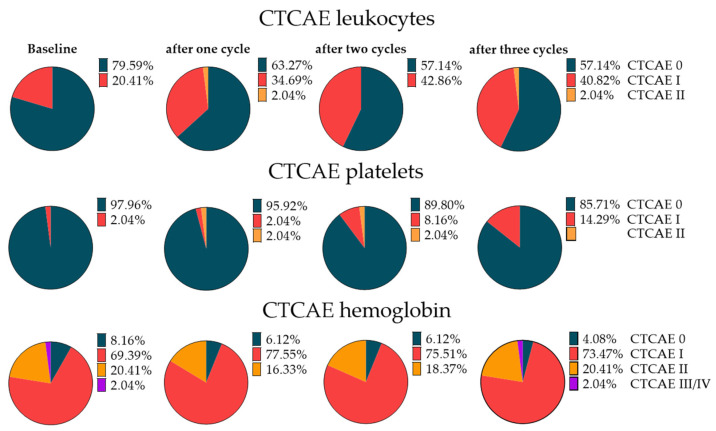
Pie charts for leukocytes, platelets, and hemoglobin classified according to CTCAE for 49 patients under radioligand therapy (RLT) before and at each cycle of therapy. Under RLT, an increasing rate of CTC I leukopenia, anemia, and thrombopenia was noted, which was more pronounced for leukocytes.

After three cycles of RLT, 1/49 (2%) showed CTCAE II and 20/49 (41%) showed CTCAE I leukocytopenia, while in the remainder (57%), no substantial changes were recorded. Regarding platelets, 7/49 (14%) showed grade I toxicity, while in the remaining cohort, the platelet count was not affected. For hemoglobin, only two new (4%) grade I toxicities occurred (Figure 4). The absolute leukocyte and platelet counts after three cycles were 5.6 ± 2.1 * 1000/µL and 215.8 ± 58.8 * 1000/µL, respectively, with a mean decrease of 15.2% and 16.6% (*p* < 0.001, respectively). Hemoglobin after three cycles was 11.0 ± 1.6 g/dL (mean decrease 4.7%, *p* < 0.05).

### 3.7. Extended Follow-Up Cohort Hematotoxicity

**Figure 5:**
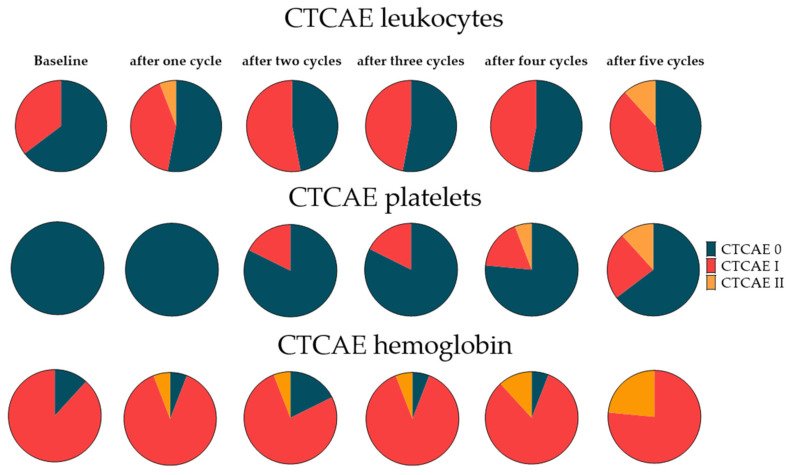
Pie charts for leukocytes, platelets, and hemoglobin for the extended follow-up cohort. Classifications according to CTCAE for leukocytes and platelets for 17 patients under radioligand therapy before and at each cycle of therapy. During long-term follow-up, an increasing rate of CTCAE I/II leukopenia and thrombopenia was noted, which again was more pronounced for leukocytes. Detailed percentages can be found in the Table S2.

Extended follow-up after the 5th cycle was available for 17 patients. Regarding leukocytes, 2/17 patients (12%) showed grade II, and 7/17 (41%) showed grade I toxicity, while in the remaining patients (47%), no significant change was recorded. Regarding platelets, 2/17 (12%) had grade II toxicity, and 4/17 (24%) showed grade I toxicity, while for the remaining subjects, the platelet count was not affected. For hemoglobin, the following toxicities were recorded: grade I toxicity in 13/17 (76%) and grade II in 4/17 (24%) (Figure 5).

**Figure 6:**
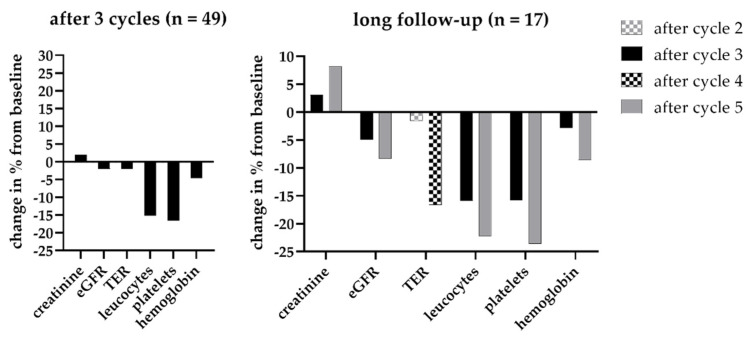
Relative changes in renal function parameters as well as leukocyte counts, platelet counts, and hemoglobin under ongoing radioligand therapy (left, after three cycles; right: after five cycles). For renal functional parameters, no substantial changes after three cycles and during extended follow-up were recorded. For leukocytes and platelets, however, a remarked decrease was already noted after three cycles, which was further enhanced after five cycles. TER = tubular extraction rate, eGFR = estimated glomerular filtration rate.

The absolute leukocyte counts showed a decrease from 6.3 ± 2.6 to 5.3 ± 1.9 * 1000/µL after three cycles (−15.9%, *p* < 0.05) and to 4.9 ± 1.9 * 1000/µL after five cycles (−22.2%, *p* < 0.001). The absolute platelet counts showed a decrease from 245.8 ± 68.1 to 206.6 ± 59.5 * 1000/µL (−15.9%, *p* = 0.008) after three cycles and to 187.9 ± 68.8 * 1000/µL after five cycles (−23.6%, *p* < 0.001). Hb decreased from 12.1 ± 1.2 g/dL to 11.8 ± 1.3 g/dL (−2.9%, *p* = 0.09) after three cycles, and to 11.1 ± 1.6 g/dL (−8.6%, *p* < 0.05) after five cycles. Figure 6 shows the relative changes in leukocyte and platelet counts and alterations of renal functional parameters during short- and long-term follow-up.

## 4. Discussion

RLT with [^177^Lu]-labeled PSMA ligands is a novel therapeutic option for patients with mCRPC and may become more established, in particular after the successful results of the VISION trial [10]. This trial was conducted with [^177^Lu]Lu-PSMA-617 and, as mentioned before, [^177^Lu]Lu-PSMA I&T and [^177^Lu]Lu-PSMA-617 are comparable in their safety profile at the meta-analytic level [11,14], but data on side effects using [^177^Lu]Lu-PSMA I&T are rather limited.

In our retrospective study with [^177^Lu]Lu-PSMA I&T, we recorded relevant nephrotoxicity in only a small fraction of patients, thereby corroborating previous results also reporting no grade III/IV nephrotoxicity in patients treated with [^177^Lu]Lu-PSMA I&T [13]. Our results are in line with a large meta-analysis, mainly analyzing retrospective data, where overall nephropathy was tabulated in up to 13%, and grade III/IV in only 1% of the patients [11]. Similar findings were observed in prospective studies using [^177^Lu]Lu-PSMA-617 [8,9,12].

Interestingly, about one-quarter of patients already had significantly elevated creatinine levels at baseline, which is in line with findings of Yordanova et al. for [^177^Lu]Lu-PSMA-617 [16]. In contrast, in our cohort, 69% of the patients already had a relevant CTCAE grade for eGFR, thereby indicating that the current CTCAE definition of “normal” renal function may not apply for the investigated cohort of especially elderly patients with end-stage mCRPC. In this regard, the classification according to CTCAE may be restricted by the fact that the impaired renal baseline status is not further considered during follow-up. Other classification systems, such as KDIGO GFR categories, may better reflect actual changes of renal function with RLT [17]. In addition, using this system, Gallyamov et al. reported three G3a cases in a mixed cohort treated with [^177^Lu]Lu-PSMA I&T and -617, which is in line with our findings of two individuals worsening from G3a to G3b under RLT [18]. Even when such sophisticated approaches such as KDIGO are applied to assess current renal functional status with RLT, alterations or re-categorizations do not frequently occur. As such, while nephrotoxicity should be discussed with patients considering RLT, it is reasonable to emphasize to them that significant effects on kidney function are uncommon. As stated before, renal impairment should not lead to categorical exclusion of patients [19].

Similar to eGFR and creatinine, renal scintigraphy-derived TER also showed only a slight decrease (median decline 4.2 mL/min/1.73 m^2^), which is lower than the loss of renal function in the Lu-PSMA trial, where patients had a mean decline of 11.7 mL/min using the GFR-reflecting [^51^Cr]-ethylenediaminetetraacetic acid three months after completion of RLT [8]. In our cohort, we applied a lower normal limit of 70% of the age-dependent normal values for TER, and only 2%–6% of the patients demonstrated impaired renal function after two cycles and 6% after four cycles, further emphasizing the high renal safety profile of [^177^Lu]Lu-PSMA I&T. Nonetheless, relative to eGFR or creatinine, renal scintigraphy plays an important role in the work-up of patients scheduled for RLT, as it provides information on split renal function [20]. Accordingly, regularly performed renal scintigraphy may identify patients with (tumor-related) obstructive nephropathies, which are at an increased risk of more severe hematologic side effects due to an increased whole-body residence time of the administered radiopharmaceutical [21].

After analyzing risk factors and correlating them with renal function during follow-up, we conclude that lower age and better initial renal function, as well as a lower Gleason score, are associated with lower rates of CTCAEs. This is in line with the findings of Yordanova, who also reported age as a risk factor for the occurrence of renal toxicity using [^177^Lu]Lu-PSMA-617 [16]. Regardless of which radiopharmaceutical is used, elderly patients should be monitored closely not to miss the development of nephropathy after RLT.

We found a substantial number (about 69% and 20%) of patients had anemia and leukopenia CTCAE I before initiation of treatment, which may indicate that the definition of CTCAE in anemia and leukopenia depends strongly on the normal values established by the analyzing lab. At our hospital, the lab sets the threshold at 14 g/dL for hemoglobin and 5.0 * 1000/µL for leukocytes, which led to a high number of initial events. Regarding the percentage of leukopenia after three cycles, our cohort presented a mild form in 43% (only grade I/II, no grade III/IV), which is slightly higher than in a previously published meta-analysis (all: 28%; grade III/IV: 4%) [11]. Again, this is most likely attributable to the aforementioned high number of initial events and the definition of CTCAE in our lab.

Regarding anemia after three cycles, 96% of the patients presented with a mild form (grade I/II), and only one patient (2%) presented with grade III toxicity, which appears higher than in the previously published meta-analysis (all: 28%, grade III/IV 8%) [11]. This may be partially explained by the high number of initial events, and the definition of CTCAE in our lab as only two patients (4%) had a “new” grade I toxicity.

The rate of CTCAEs for platelets was low with 14% (only grade I) after three cycles but increased to 36% grade I/II after five cycles (with no grade III/IV). These values are comparable to previously published results in a meta-analysis (all: 23%; grade III/IV: 3–4: 4%) [11], but slightly lower than when compared to recent results of prospective trials using [^177^Lu]Lu-PSMA-617, e.g., in the Lu-PSMA trial (III/IV: 10%) [8], the TheraP-trial (III/IV: 11%) [9] or the VISION trial (III/IV: 7.9%) [10]. Using [^177^Lu]Lu-PSMA I&T, Heck et al. reported on grade III/IV thrombocytopenia in only 4% [13], which is in line with our findings of no ≥ III CTCAE events. Such observed differences in the rates of serious side effects may be partially explained by the use of different compounds, but we think that the number of treatment cycles, a selection bias (patients with earlier termination of therapy were not considered), the time points of toxicity evaluation, and especially different amount of administered activities may further explain the discordant results (our cohort: 6.0 GBq every 8 weeks, Lu-PSMA: 7.5 GBq every 6 weeks, TheraP: 6.0–8.5 GBq every 6 weeks, VISION: 7.4 GBq every 6 weeks) [7,9,10]. As such, treatment protocol may have a significant impact on the rate of thrombocytopenia. Another aspect of occurrence of side effects might be the duration of therapy, with concomitant increasing absorbed doses to the bone marrow. From RLT with [^177^Lu]Lu-PSMA-617, it is known that hematologic side effects occur with an acceptable overall frequency and are often reversible [22].

We were able to analyze a smaller fraction of our cohort with an extended follow-up that underwent at least five cycles of therapy and observed increasing nephrotoxicity and, in particular, an increased rate of hematological events, with an absolute decrease in platelet and leukocyte counts of >15%. Future studies investigating [^177^Lu]Lu-PSMA I&T may also consider absorbed doses, e.g., by analyzing post-therapeutic whole-body single-photon imaging at multiple time points [23].

Our study has several limitations: This retrospective study includes a small sample size, especially for the long follow-up cohort. Treatment and follow-up were not completely standardized, but the therapy was conducted following the same protocol over years and in accordance with current guidelines. In addition, relevant possible adverse events were registered on a regular basis. Unfortunately, we can not provide detailed information on lymphopenia or neutropenia, as these values were not recorded regularly. Future studies investigating the hematologic side effects of [^177^Lu]Lu-PSMA I&T should also include laboratory values related to lymphopenia or neutropenia. In addition, correlation of pre-therapeutic tumor burden with detailed hematological laboratory values during follow-up would then be possible [24].

## 5. Conclusions

Prior to treatment with [^177^Lu]Lu-PSMA I&T, a substantial fraction of patients already showed impaired renal function, anemia, and reduced leukocyte counts. Under ongoing RLT, additional severe nephrotoxicity or hematotoxicity was uncommon. No new severe (grade III/IV) toxicities occurred after three cycles, but low-grade nephro- and hematotoxicity relatively occurred more often after five cycles, which may be linked to either increasing absorbed doses and/or time-dependent factors of toxicity.

## Figures and Tables

**Table 1:** Patient characteristics.

Variable	Mean ± SD
Age at first cycle of PSMA RLT (years)	71.2 ± 8.4
Treatment cycles per patient	4.8 ± 1.8
Administered cumulative activity (GBq)	17.8 ± 1.5
Gleason score	8 ± 1 *
Previous Treatments	Patients (%)
Radical prostatectomy	24 (49.0%)
Primary radiation therapy to the prostate	9 (18.4%)
Adjuvant radiation therapy	12 (24.5%)
Salvage radiation therapy	6 (12.2%)
Antihormonal treatment	49 (100%)
Enzalutamide	38 (77.6%)
Abiraterone acetate	35 (71.4%)
Previous chemotherapy	28 (57.1%)
Clinical Risk Factors for Nephrotoxicity	Patients (%)
Pre-existing renal disease	12 (24.5%)
Arterial hypertension	32 (65.3%)
Diabetes mellitus	8 (16.3%)
Nephrotoxic drugs	34 (69.4%)
Tumor burden	
Extent of tumor	
- 3 lesions	1 (2.0%)
- 4–10 lesions	6 (12.2%)
- >10 lesions	42 (85.7%)
- Thereof with diffuse tumor burden	12 (24.5%)
Organ system	
- Bones	46 (93.9%)
- Lymph nodes	35 (71.4%)
- Local	17 (34.7%)
- Liver	3 (6.1%)
- Lung	7 (14.3%)
Baseline Laboratory Values	Mean ± SD
PSA (μg/L)	374.1 ± 623.1
eGFR (mL/min/1.73 m^2^)	76.5 ± 20.1
^99m^Tc-MAG3-derived TER (mL/min/1.73 m^2^)	213.3 ± 61.5 *

PSA = prostate-specific antigen, eGFR = estimated glomerular filtration rate, MAG3 = [^99m^Tc]Tc-mercaptoacetyltriglycine, TER = tubular extraction rate. * available in *n* = 42.

## Data Availability

The main data presented in this study are available in the article. Detailed information about the image analysis or the overall survivals of the subjects presented in this study is available on reasonable request from the corresponding author.

## Supplementary Materials

The following are available online at https://www.mdpi.com/article/10.3390/cancers14030647/s1, Table S1: Distribution of patients in the extended cohort (*n* = 17) in the different CTCAE groups for renal toxicity and distribution of the patients according to KDIGO. Table S2: Distribution of patients in the extended cohort (*n* = 17) in the different CTCAE groups for hematotoxicity.
