# Supplementary material for: Hematotoxicity and Nephrotoxicity in Prostate Cancer Patients Undergoing Radioligand Therapy with [177Lu]Lu-PSMA I&T
Source: Cancers (Basel). 2022 Jan 27;14(3):647. doi: 10.3390/cancers14030647 (PMC8833540; doi:10.3390/cancers14030647)
Supplement: Supplementary file 1 [file cancers-14-00647-s001.zip › cancers-1472620-supplementary.pdf]

cancers MDPI

Table S1: Distribution of patients in the extended cohort (n=17) in the different CTCAE groups for renal toxicity and distribution of the patients according to KDIGO.

		**Baseline**	**%**	**After one cycle**	**%**	**After two cycles**	**%**	**After three cycles**	**%**	**After four cycles**	**%**	**After five cycles**	**%**
**CTCAE Creatinine**	CTC 0°	14	82.4	15	88.2	14	82.4	14	82.4	13	76.5	12	70.6
	CTC I°	2	11.8	2	11.8	3	17.6	3	17.6	3	17.6	4	23.5
	CTC II°	1	5.9	0	0.0	0	0.0	0	0.0	1	5.9	1	5.9
	CTC III°/IV°	0	0.0	0	0.0	0	0.0	0	0.0	0	0.0	0	0.0
**CTCAE eGFR**	CTC 0°	4	23.5	4	23.5	5	29.4	5	29.4	4	23.5	5	29.4
	CTC I°	10	58.8	11	64.7	9	52.9	8	47.1	10	58.8	9	52.9
	CTC II°	3	17.6	2	11.8	3	17.6	4	23.5	3	17.6	3	17.6
	CTC III°/IV°	0	0.0	0	0.0	0	0.0	0	0.0	0	0.0	0	0.0
**KDIGO**	G1	4	23.5	4	23.5	5	29.4	5	29.4	4	23.5	5	29.4
	G2	10	58.8	11	64.7	9	52.9	8	47.1	10	58.8	9	52.9
	G3a	2	11.8	2	11.8	2	11.8	3	17.6	1	5.9	0	0.0
	G3b	1	5.9	0	0.0	1	5.9	1	5.9	2	11.8	3	17.6
	G4	0	0.0	0	0.0	0	0.0	0	0.0	0	0.0	0	0.0

Table S2: Distribution of patients in the extended cohort (*n*=17) in the different CTCAE groups for hematotoxicity.

		**Baseline**	%	**After one cycle**	%	**After two cycles**	%	**After three cycles**	%	**After four cycles**	%	**After five cycles**	%
**CTCAE leukocytes**	CTC 0°	11	64.7	9	52.9	8	47.1	9	52.9	9	52.9	8	47.1
	CTC I°	6	35.3	7	41.2	9	52.9	8	47.1	8	47.1	7	41.2
	CTC II°	0	0.0	1	5.9	0	0.0	0	0.0	0	0.0	2	11.8
	CTC III°/IV°	0	0.0	0	0.0	0	0.0	0	0.0	0	0.0	0	0.0
**CTCAE platelets**	CTC 0°	17	100	17	100	14	82.4	14	82.4	13	76.5	11	64.7
	CTC I°	0	0.0	0	0.0	3	17.6	3	17.6	3	17.6	4	23.5
	CTC II°	0	0.0	0	0.0	0	0.0	0	0.0	1	5.9	2	11.8
	CTC III°/IV°	0	0.0	0	0.0	0	0.0	0	0.0	0	0.0	0	0.0
**CTCAE hemoglobin**	CTC 0°	2	11.8	1	5.9	3	17.6	1	5.9	1	5.9	0	0
	CTC I°	15	88.2	15	88.2	13	76.5	15	88.2	14	82.4	13	76.5
	CTC II°	0	0	1	5.9	1	5.9	1	5.9	2	11.8	4	23.5
	CTC III°/IV°	0	0	0	0	0	0	0	0	0	0	0	0
